# The Impact of Acquired Genetic Abnormalities on the Clinical Translation of Human Pluripotent Stem Cells

**DOI:** 10.3390/cells10113246

**Published:** 2021-11-19

**Authors:** Alexander Keller, Claudia Spits

**Affiliations:** Research Group Reproduction and Genetics, Faculty of Medicine and Pharmacy, Vrije Universiteit Brussel (VUB), 1090 Jette, Belgium

**Keywords:** pluripotent stem cells, embryonic stem cells, induced pluripotent stem cells, genomic instability, differentiation, clinical translation, copy number variations, mosaicism

## Abstract

Human pluripotent stem cells (hPSC) are known to acquire chromosomal abnormalities, which range from point mutations to large copy number changes, including full chromosome aneuploidy. These aberrations have a wide-ranging influence on the state of cells, in both the undifferentiated and differentiated state. Currently, very little is known on how these abnormalities will impact the clinical translation of hPSC, and particularly their potential to prime cells for oncogenic transformation. A further complication is that many of these abnormalities exist in a mosaic state in culture, which complicates their detection with conventional karyotyping methods. In this review we discuss current knowledge on how these aberrations influence the cell state and how this may impact the future of research and the cells’ clinical potential.

## 1. On Stem Cells

Stem cells are defined by their ability to self-renew and differentiate into more specialized cell types. The first use of the term stem cell, or “stammcelle”, was to describe a fertilized egg in the 1860s by Ernst Haeckel; however, it was the discovery of the hematopoietic stem cell in 1963 that popularized the term [1,2], ushering in an era of scientific advancement. Stem cells come in a variety of types—totipotent, pluripotent, multipotent, and unipotent, depending on the range of cells they can differentiate into. Pluripotent stem cells (PSC), the focus of this review, are one step below totipotent cells and can differentiate into all cell types of the three germ layers, and include embryonic (ESC) and induced pluripotent stem cells (iPSC). The potential applications of human pluripotent stem cells (hPSC) are many, but the most important fall into the fields of drug discovery, disease modeling, and regenerative medicine. At their core, each of these applications takes advantage of the differentiated derivatives of hPSC, and the relative ease with which they can be generated.

In drug discovery and toxicity screening, researchers can bypass the need for animal models, avoiding issues of interspecies differences that often lead to failed translation to humans as well as the ethical issues related to animal research. hPSC have demonstrable advantages over primary cells as well. Complications related to the cells’ availability and culture are made obsolete. Reprogramming somatic cells offers further advantages through the generation of patient-derived iPSCs, opening up the possibility of “patient-derived disease-in-a-dish models”, whereby cell types with disease-specific phenotypes are readily created. These models offer a more realistic source material for drug screening and have led to the discovery and subsequent clinical trials of drugs for progressive supranuclear palsy (Gosuranemab, currently in phase II), amyotrophic lateral sclerosis (retigabine, currently in phase II; bosutinib, currently in phase I; ROPI, phase I/IIa), and spinal muscular atrophy (RG7800, entering phase II) [3,4,5]. 

Building on the concept of the disease-in-a-dish, hPSC have potential for modeling human genetic disorders. By establishing hPSC lines with specific genetic disorders, it is possible to study the molecular mechanisms that drive the disease and develop new treatment strategies. While modeling monogenic disorders has generally been more successful than the study of complex multigene disorders, there are numerous models of both types—for example, monogenic disorders such as fragile X syndrome [6] and myotonic dystrophy [7,8], and complex disorders such as autism spectrum disorder [9] and schizophrenia [10].

Perhaps the most important biomedical application for hPSC lies in their potential to act as a cell source for regenerative medicine. With their potential to differentiate into all cell types of the human body, it is theoretically possible to generate viable replacement tissues for various degenerative disorders. The most prominent hPSC-derived cell type currently in clinical trials is retinal pigmented epithelium (RPE) cells for the treatment of age-related macular degeneration [11], with eight ongoing clinical trials and several already completed. Promising work with oligodendrocytes for the treatment of spinal cord injury [12], pancreatic beta cells for the treatment of type 1 diabetes [13], and dopaminergic neurons for the treatment of Parkinson’s disease is also ongoing [14]. Over 27 clinical trials are either ongoing, or completed, with generally positive outcomes demonstrating safety and limited but promising levels of efficacy [15] (www.clinicaltrials.gov (accessed on 15 September 2021)). 

## 2. De Novo Copy Number Variation in hPSC

Though hPSC are typically genetically euploid after derivation, they are known to acquire genetic abnormalities in the form of copy number variations (CNVs) with time in culture. The first reports on chromosomal abnormalities in hESC date from 2004, initially only in chromosomes 12 and 17 [16], but a large collaborative study published in 2011 made it clear that these aberrations were present across the entire genome in lines worldwide [17]. Although takeover is typically observed after extended culture in late-passage hPSC, reports vary, with takeover seen in as few as five passages in some cell lines, while others remain chromosomally normal for 200 passages [18]. Initially, most identified genetic aberrations were whole chromosome gains, with a few chromosome losses and some small segmental aberrations. Subsequent improvements in screening methods built on these findings, demonstrating that segmental gains and losses were also very common [19]. 

Importantly, these aberrations are present in both hESC and hiPSC, indicating a common mechanism of formation and culture take-over [20]. While aberrations are found across the entire genome, gains of 1, 12, 17, and 20 are the most frequent, and losses of chromosomes 18q and 10p occur to a lesser extent. This strongly suggests that an underlying mechanism gradually leads to takeover of these specific aberrations, driving a form of natural selection in a dish. Indeed, driver genes that improved cell survivability were quickly identified in many recurrent aberrations. The anti-apoptosis factor BCL-xL, encoded by *BCL2L1*, was the first driver gene to be identified, providing a survival advantage to cells with a gain at 20q11.21 [21,22]. This was soon followed by the identification of a proliferative advantage in hPSC with trisomy 12, partially resulting from overexpression of the pluripotency-associated gene *NANOG* [23]. The specific driver genes associated with the remaining recurrent chromosomal aberrations are yet to be identified; however, strong candidates have been proposed [24]. Located within the minimal region of gain on chromosome 1q [19] is the gene *MDM4*, a p53 suppressor, which has been proposed to increase the threshold to reach apoptosis [25]. Similarly, another antiapoptotic gene, *BIRC5*, located at 17q25.3, has been proposed as the primary driver for gains of chromosome 17 [26], though a proliferative advantage has been seen in the cells as well [27]. It has also been demonstrated that some chromosomally hPSC not only outcompete (through reductions in apoptosis and increased proliferation), but also directly induce apoptosis in chromosomally diploid cells in the culture, facilitating an even faster takeover.

Interestingly, despite the interest in and proposed advantages associated with naïve hPSC, the genomic integrity of these cells remains largely unknown. Scattered reports on naïve conversion have indicated that naïve hPSC also acquire chromosomal aberrations over time in culture, with reports of trisomies of 3, 7, 12, 20, and X [28,29,30]. The overlap in gains of chromosomes 12 and 20, and, less commonly, 3, between naïve and primed hPSC is notable and indicates a common mechanism of selection between the two states. 

## 3. On the Origin of Chromosomal Abnormalities

The mechanisms driving the high instance of chromosomal abnormalities in hPSC are still poorly understood, but are generally thought to be the result of suboptimal culture techniques and high levels of DNA damage compared to somatic cells [31,32]. This increase in DNA damage is in part due to high replication stress in the cells [33,34]. Replication stress is known to lead to replication fork stalling, which can result in DNA double-strand breaks [35] and subsequent chromosomal rearrangements as a result of errors in repair [36]. This mechanism may be worsened by hPSCs’ ability to bypass key cell cycle checkpoints, which explains their characteristically short cell cycle [37,38] Furthermore, it has been demonstrated that medium acidification in cultures with mouse feeders leads to increased DNA damage and chromosomal abnormalities, potentially aggravating the issue further [39,40]. Strikingly, hPSC that have already acquired one chromosomal abnormality may be predisposed to acquire them more readily, as aberrant cells have an increase in chromosome condensation defects, which leads to additional replication fork stalling and collapse [34]. This may help explain why cell lines with multiple chromosomal aberrations are so frequently identified. Medium acidification and replication fork stalling can be relieved by more frequent media changes and the addition of nucleosides to the culture media, respectively [33,34,35,36,37,38,39] indicating that improvements to culture methods may help alleviate the issue.

## 4. Point Mutations

In contrast to the relatively high frequency of chromosomal aberrations, the mutation rate in hPSC has been reported to be lower than what is seen in somatic cells [41,42]. In line with this, hPSC have been reported to have a highly active DNA repair system [43], with a preference for homologous recombination over nonhomologous end joining, which leads to more accurate repair after double-strand breaks [44]. Furthermore, hPSC also utilize a secondary nonhomologous end joining mechanism that supports high-fidelity repair. 

However, it has been reported that hPSC acquire point mutations, including recurrent dominant negative *TP53* mutations, which provide a selective advantage [45,46]. This signifies that, while the mutation rate is low, the same selective pressures that enrich for recurrent chromosomal aberrations can also enrich for mutations that provide a survivability advantage. The mutational load in cancer-related genes between naïve and primed appears to be largely similar [47], if not higher in primed hPSC, where additional point mutations were identified in the cancer-related genes *EGFR*, *PATZ1*, and *CDK12* [48]. Naïve hPSC also carry both *TP53* and *CDK12* mutations, indicating, as with CNVs, a similar mechanism of enrichment for mutations that provide a survival or growth advantage.

## 5. Altered Differentiation Propensity as a Result of Genetic Abnormalities

The differentiation capacity of hPSC is crucial to their potential application. However, significant variation exists in the differentiation capacity of the cells as a result of a wide range of factors, including the multitude of CNVs found in the cells (recently reviewed in [49]). Until recently, the majority of findings pertaining to the impact of CNVs on hPSC differentiation have been based on single cell lines, did not control for genetic background, and/or failed to identify a mechanism of differentiation impairment. Two recent reports by our group bridge these shortcomings by using multiple isogenic cell lines to evaluate the impact of gains of 12p and 20q, two of the most common recurrent CNVs in hPSCs, on differentiation [50,51]. In Keller et al., we described a general reduction in differentiation capacity in cells carrying a 12p13.31 gain. Furthermore, we demonstrated that residual pluripotent cells regularly form during hepatic progenitor differentiation in these cells. This is driven directly by the overexpression of *NANOG* and *GDF3*, which impair the dissolution of the pluripotent state and form a positive feedback loop that maintains the cells in an undifferentiated state in differentiation-promoting conditions. In Markouli et al. we demonstrate that gains of 20q11.21 cause a near complete loss of neurectoderm differentiation capacity as a result of altered TGFβ and SMAD signaling. 

## 6. Residual Pluripotent Cells and Tumorigenicity

Residual pluripotent cells are one of the greatest risks to the safe clinical translation of the cells as a result of their ability to form teratomas [52]. Several case studies involving individuals seeking unproven treatments through stem cell tourism have highlighted the potential for serious complications [53,54,55,56]. In one case study, a patient developed glioproliferative lesions of non-host origin after an hESC transfer, clearly demonstrating the real-world risk of inadequately controlled cell therapy products. While significant work has been done to detect and purify these cells, little is known about how they form and are maintained in conditions that otherwise promote differentiation [57,58,59,60,61,62,63,64,65,66,67,68,69,70]. 

## 7. The First Hit Hypothesis

**Figure 1:**
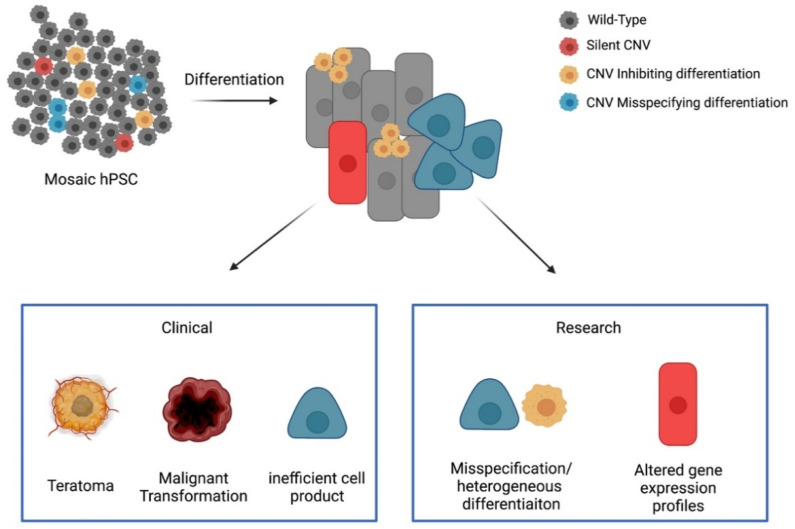
Overview of the potential impacts of chromosomal abnormalities on research and clinical outcomes: Any given population of hPSCs may be present as a mosaic (multicolored starting undifferentiated population). Once differentiated, each of these cells has the potential to negatively influence the differentiation outcome (heterogeneity or gene expression profiles) or be the source of a future negative outcome (teratoma or malignant transformation) (Created with BioRender.com accessed on 3 November 2021).

A second concern is that genetic abnormalities could lead to the transplanting of transformed cells or cells that can later transform, possibly after undergoing other spontaneous mutations in vivo. Transformed cells of this type would be significantly harder to detect than residual pluripotent cells as they may be phenotypically silent during differentiation. Furthermore, a lack of inherent differences between transformed and nontransformed cells may make them difficult to target using similar methods for residual pluripotent cell removal. Many of the recurrent chromosomal aberrations seen in hPSC are also associated with known cancers. Gains of chromosome 1 have been well studied in brain tumors, B cell lymphoma, Wilms tumors, intercranial ependymomas, and multiple myeloma, where they are associated with a poor prognosis [71,72,73,74,75]. Gains of chromosomes 12 and 17 are associated with testicular germ cell tumors [76,77], with gains of 17 also associated with neuroblastomas [78]. Gains of chromosome 20q are associated with colorectal cancer [79] and cervical cancer [80], and are generally associated with tumorigenic transformation [81,82].Losses of 18q are associated with colorectal carcinoma [83]. Furthermore, chromosomal aberrations in hPSC are associated with an increased tumor initiation capacity in the teratoma assay and their teratomas display neoplastic properties [84,85]. Tumors resulting from hPSC-derived products containing one or more of these CNVs could therefore be malignant in nature (an overview of the potential outcomes resulting from chromosomally abnormal hPSC can be found in Figure 1).

## 8. Mosaicism in hPSC Cultures

While a majority of hPSC cultures are reported to be genetically diploid, most studies to date have only evaluated the genetic content of bulk populations of cells. It is now clear that hPSC carry mosaic chromosomal abnormalities. Work by our group and others has demonstrated that hPSC cultures are highly mosaic and carry a significant load of chromosomal aberrations that cannot be detected by bulk analysis [20,39,86,87]. These studies indicate that as many as 20% of cells carry at least one megabase chromosomal aberration, which is likely an underestimation due to current limitations in the resolution of single-cell karyotyping. The CNVs seen at the single cell level are distributed throughout the genome, with an increased abundance in subtelomeric regions, without specific enrichment for known recurrent aberrations. This suggests that chromosomal aberrations are random, and that takeover only occurs when a region that is gained or lost provides a culture advantage. Most CNVs do not provide a survivability advantage in the undifferentiated state, and so will likely remain in drift or disappear from the culture. However, even within the relatively small numbers of single cells tested in different studies from our lab [39,86,87], we have identified low-grade mosaic gains of 1q, 12p, and 20q, all of which are expected to take over the culture. Given the high prevalence of chromosomal abnormalities in hPSC worldwide [17,19], it is likely the mosaicism seen by us can be extrapolated to the field as a whole. 

There are several potential repercussions that could result from mosaicism in hPSC. The impact on research outcomes is one consideration. Given that a majority of research is performed on bulk populations, researchers may be unknowingly working with a highly mosaic culture, which could have negative implications for experimental findings. This general concept has been leveraged by research groups who have used publicly available datasets to retrospectively demonstrate novel findings from mosaic cultures. For example, the discovery of dominant negative mutations in *TP53* in hPSC was confirmed in publicly available RNAseq datasets, where a number of mosaic *TP53* mutations could be identified [46]. Recently, a similar approach was used to identify additional mosaic cancer mutations in hPSC [48]. These datasets demonstrate the existence of mosaic cultures in published work. It could be argued that both of these examples were used to identify coding mutations, which would have been more difficult to identify by the original researchers than a CNV. However, high-resolution reporting of the genetic content of cell lines remains uncommon in hPSC research, and evaluation of culture mosaicism is rarely, if ever, reported. As such, the likelihood of past and future findings being performed on mosaic cell lines is high. Though the impact of mosaic cultures is largely unknown, recurrent CNVs have been shown to have a significant influence on gene expression patterns [23,51,88]. Furthermore, it is possible that the influence a given mosaic CNV has on a culture may be disproportionate relative to the level of mosaicism. One report has indicated that genetically normal hPSC cocultured with hPSC carrying a trisomy 12 or a gain of 20q11.21 led to the genetically normal hPSC taking on the mutant cells’ characteristics, shifting the cells’ molecular network to a more neoplastic phenotype [89]. A more recent report indicated that chromosomally abnormal hPSC can also induce apoptosis in genetically normal cells through direct contact during culture [90]. Taken together, it is clear that suboptimal outcomes are expected from mosaic cultures. It is important to note that these observations only consider the potential outcomes in the undifferentiated state and with known mutations. 

It is also possible that mosaic hPSC negatively impact differentiation, beyond simply having a mixed population of cells. Coculture of hPSC with primary cells has previously been used to improve differentiation protocols [91,92], and more recently, researchers have suggested that cells that do not go on to form iPSC during reprogramming are perhaps equally important to the conversion process [93]. Using scRNAseq, they identified and validated the paracrine factor GDF9, secreted from a subpopulation of cells that formed midway through the process, as significantly improving reprogramming efficiency when added exogenously, but only within a specific window of time. These reports demonstrate that paracrine effects can be crucial to correct cell specification. It is therefore equally possible, given the importance of factor timing and concentration, that uncontrolled paracrine effects stemming from mosaic CNV-induced heterogeneity may negatively impact differentiation. It is conceivable, then, that a mosaic culture could have a more widespread impact on bulk populations than the percentage of the total population they represent would suggest, potentially confounding research findings.

Another equally important consideration is the potential for mosaic cultures to have as yet unknown consequences on clinical translation. It is clear that mosaic CNVs can be spread across the entire genome, and while some may be recurrent CNVs that have been studied, at least to a degree, de novo CNVs are poorly described, if at all. It is possible that a clinical product derived from a mosaic population will carry some cells that have a mutation that could prove to be tumorigenic. While many of the most recurrent CNVs in hPSC are associated with cancer, these CNVs are known, and targeted screening methods could be developed to identify them. Unique, randomly distributed CNVs are more difficult to identify, while still presenting a significant risk for potential malignant transformation. As an example, in [87], de novo CNV-carrying cells were identified that carried segmental gains in chromosome 3 and loss of chromosome 7, neither of which is part of the common set of recurrent CNVs in hPSC, and each is in a region associated with cancer [94,95]. We also identified a majority gain of the p-arm of chromosome 7. Given that glioblastomas often begin their transformation with the acquisition of gains of chromosome 7, followed by losses of chromosome 10 [96], this cell, if transplanted as an oligodendrocyte, for example, may already be undergoing a malignant transformation. 

Similarly, residual pluripotent cells resulting from a mosaic population with cells carrying gains of 12p could lead to the formation of a teratoma tumor. Given that trisomy 12 hPSC have been described as forming teratomas with neoplastic features, from which undifferentiated cells can be extracted [23,84], this significantly increases the risk they present. The mechanism we present for the formation of residual pluripotent cells is not dependent upon a pure population of 12p cells; as such, even a small population of 12p cells mixed within a culture have the potential to lead to residual pluripotent cell contamination in a clinical product and a negative impact on hPSC research.

## 9. Strategies for Genetic Screening in hPSC Lines

During recent discussions as part of the International Stem Cell Initiative and the International Stem Cell Banking Initiative, the issues of genomic integrity and safety were acknowledged as central to the challenges faced in the field [97]. However, international consensus is lacking on what the best approach is to tackle the issue. Furthermore, to date there are no known solutions to this issue, as hPSC acquire de novo CNVs very quickly in culture [87]. Screening is also challenging, as the mostly commonly used methods, such as Quantitative Real-time PCR (qPCR), G-Banding, FISH, array-based comparative genome hybridization (aCGH), and whole-genome sequencing (WGS), are commonly accepted to only detect CNVs present at a fraction greater than 10% [19]. Given the need for hundreds of millions to billions of cells for effective therapies in regenerative medicine [98], it will be impossible to screen every cell that is destined for transplantation, leaving some level of inherent risk in all transplantations. Furthermore, each of these techniques has inherent limitations: for example, ddPCR/qPCR and FISH are limited to a selection of targets, G-banding cannot detect small aberrations, and aCGH and WGS cannot detect rearrangements (Table 1).

While, to date, no adverse outcomes have been observed in clinical trials involving hPSC-derived products, the first human trial using the cells at the RIKEN Institute in Japan was suspended as a result of the identification of three single-nucleotide variants (SNVs) and three CNVs, one SNV of which was listed in a database of known cancer somatic mutations [99]. It has been suggested to use the COSMIC (Catalogue Of Somatic Mutations In Cancer) database and the Shibata list from the Pharmaceuticals and Medical Devices Agency in Japan (http://www.pmda.go.jp/files/000152599.pdf#page=8 (accessed on 15 November 21)) to predict the functionality of these variants [100,101,102]. This approach has some shortcomings, however: these databases not only contain driver mutations, but also variants that do not contribute to tumor development or progression [103]. Interestingly, while WGS was required for the canceled RIKEN clinical trial, subsequent clinical trials on Parkinson’s disease removed this requirement due to issues related to interpretation [52]. While cancer-associated mutations originating from the hPSC source material would be quickly identified, mutations originating from hPSC themselves may be easily missed if present as a mosaic. Furthermore, defining the cancer risk associated with a specific mutation is challenging, and no consensus has been reached on the subject when defining real-world individual mutations. Defining genetic normalcy or acceptable risk as part of clinical translation remains a challenge. Indeed, there may be no such thing as “genetically normal,” as CNVs have been observed across multiple tissues in healthy individuals [104] and the sequencing of single human neurons reveals high levels of mosaicism as well [105]. The 1000-genomes project has further solidified this notion, identifying significant genetic variance between individuals [106].

## 10. Conclusions

Since their initial discovery, our understanding of the biology of hPSC has progressed significantly, with expansion in the number of cell types available for differentiation resulting in immense progress in the field as a whole. Simultaneously with this progress has been an increase in awareness of the challenges faced by genomic instability in the cells. 

However, it is clear that these hurdles are not insurmountable and improved understanding of hPSC biology will lead to innovative solutions to the issues presented. It should be noted that clinical trials involving hPSC have, up to now, shown the cells to be generally safe [11,107,108,109], and future clinical trials will continue to build upon the advances made so far. This raises the question of the real-world risk associated with hPSC-derived clinical products. Significant genetic diversity already exists within the population, with copy number variations being reported across multiple different tissue types [110,111,112,113,114], and it has been reported that individuals can carry a significant mutational burden in cancer-related genes without them becoming pathogenic [115,116]. Furthermore, many cancers have been reported to have highly specific cells of origin [117]. A mutation or CNV in a cancer-causing gene may not ultimately lead to tumor formation if acquired in a cell type that is not associated with the specific cancer. Terminal differentiation of hPSC-derived products also reduces the overall risk, as malignant transformations tend to be associated with progenitor-stage cell types. One potential strategy would be to screen for cancer-specific drivers only in the cell types they can generate from, and not every cancer-related mutation in every cell type. In any event, while the potential for a tumorigenic event may ultimately be inevitable, novel strategies for dealing with the problem continue to develop. For example, a future clinical trial has proposed using HLA-mismatched hPSC in combination with immunosuppression; in this way, the immune system can be leveraged in the event of an adverse outcome [52]. Furthermore, improvements in inducible suicide genes continue [118], as do cell encapsulation strategies often used with pancreatic islets, for example. While initial efforts to identify markers of transformed hPSC ultimately did not prove successful [119,120], this remains an open question, one that modern single-cell technologies could explore further. It is clear then that, while it may not be possible to screen every cell prior to transplantation, combining targeted screening with removal strategies is likely optimal. 

Outside of the potential for tumor formation, a growing body of evidence supports the fact that chromosomally abnormal hPSC have a direct impact on differentiation and this, in turn, can have a direct impact on research outcomes. In combination with culture mosaicism and a lack of screening, it is possible that previously published work was performed on hPSC with a recurrent CNV, potentially explaining the inconsistencies between published works or findings that were unable to be reproduced. Even in cell lines that were screened by banding techniques, small segmental gains may have been missed. However, this cannot be easily tested, and in any event, standard banding techniques still detect a majority of the CNVs seen in hPSC [19]. As the cost and ease of screening continue to improve, more routine testing for recurrent CNV should become more common, alleviating this issue, at least in part.

As our understanding of genomic stability improves, methods could be found to decrease its frequency as well. For example, the addition of nucleosides to a culture medium has been demonstrated to partially alleviate replication stress [33], and targeted small-molecule-induced death of chromosomally abnormal cells has also previously been demonstrated [23,121]. If built upon, these findings could be used to improve culture methods by routinely purifying cell cultures and reducing the instance of de novo CNV formation.

## Figures and Tables

**Table 1:** Methods for genetic screening of hPSC cultures.

Technique	Advantages	Disadvantage
G-Banding	Low cost, can identify chromosomal rearrangements and mosaicism (>10% with 20–30 spreads)	Low resolution (5 Mb)
FISH	Low cost, rapid, detects low-grade mosaicism (<5%)	Targeted regions only, cannot establish breakpoints
qPCR	Speed and accessibility, mosaicism > 10%	Targeted regions only, cannot establish breakpoints
ddPCR	Mosaicism > 5%	Requires highly specialized equipment, targeted regions only, cannot establish breakpoints
aCGH and shallow WGS	High resolution, mosaicism depending on platform-typically > 10–15%	No balanced chromosomal rearrangements
WGS	Very high resolution	Cost, time, data interpretation, specialized equipment
Single-cell WGS sequencing	Mosaicism (lower limit based on number of cells sequenced)	Cost, time, low resolution, specialized equipment

FISH: fluorescent in situ hybridization. qPCR: quantitative real-time PCR. ddPCR: digital droplet PCR. aCGH: array-based compared genomic hybridization. WGS: whole-genome sequencing.
